# Fighting over defense chemicals disrupts mating behavior

**DOI:** 10.1093/beheco/arab117

**Published:** 2021-12-31

**Authors:** Sarah Catherine Paul, Caroline Müller

**Affiliations:** Chemical Ecology, Bielefeld University, Universitätsstr. 25, 33615 Bielefeld, Germany; Chemical Ecology, Bielefeld University, Universitätsstr. 25, 33615 Bielefeld, Germany

**Keywords:** conflict, dyadic contests, fighting behavior, chemical defense, mating behavior

## Abstract

Studies on intraspecific contest behavior predominantly focus on contests between individuals of the same sex, however contest behavior is also expected to occur between individuals of the opposite sex including possible mates. Here we investigate potential trade-offs between mating and fighting behavior in the turnip sawfly (*Athalia rosae*). Adults of this species collect chemical defense compounds (clerodanoids) directly from plants but also indirectly by nibbling on conspecifics that have already obtained clerodanoids, a highly aggressive behavioral interaction. An *A. rosae* individual without clerodanoids may therefore be the potential mate or attacker of an individual of the opposite sex that has gained clerodanoids. To test the effect of clerodanoids on agonistic and mating behavior we paired females and males with or without clerodanoid access in a two-way factorial design. We show that asymmetrical clerodanoid acquisition between female-male pairs causes an increase in agonistic nibbling behavior, irrespective of sex, and moreover that conflict between individuals delays mating behavior. Our study highlights the importance of investigating agonistic intersex interactions, which can occur when adults are able to acquire valuable non-reproductive resources from a potential partner.

## Teaser

Fighting over defense chemicals can disrupt potential mating opportunities when you compete with a member of the opposite sex. In the turnip sawfly, individuals acquire defense chemicals from plants, but they can also aggressively ‘steal’ them from each other. Here we show that this aggressive behavior occurs even between potential mates, disrupting mating behavior.

## INTRODUCTION

Conflict between individuals of the same species is part of the fabric of animal lives, shaping their life histories and influencing fitness (Hardy and Briffa 2013). These agonistic interactions occur over resources that are limited or which vary in quality, such as food (Gruber et al. 2016), territories (Suriyampola and Eason 2015), and protection from predators (Tricarico and Gherardi 2010). The occurrence of a dyadic fight depends on the costs and benefits of the interaction for each individual (Maynard-Smith and Price 1973; Enquist and Leimar 1987) and its outcome is determined by an interplay between the physical ability (Resource Holding Potential; RHP) and the motivation (Resource Value; RV) of each individual to win the fight (Briffa and Elwood 2000; Stockermans and Hardy 2013). The large majority of research on intraspecific dyadic contests focuses on conflict between individuals of the same sex (Andersson 1994; Stockley and Campbell 2013), however a raft of intersex interactions also occur in nature (e.g. Briffa and Dallaway 2007). Such contests may add an extra dimension to the matrix of factors influencing not only an individual’s decision to fight but fight outcome. For example, an opponent with a valuable non-reproductive resource may at the same time be a potential mate (i.e. a reproductive resource), setting up an interesting trade-off between mating and fighting, but such potential trade-offs remain little explored.

Agonistic intersex interactions may result in the loss or reduced likelihood of mating with an opposing individual of the opposite sex. Outside of the potential loss of resources by the defender, fighting also incurs significant energetic costs (Hack 1997) and increased predation risk (Jakobsson et al. 1995; Baker et al. 1999). Therefore, if an individual attacks a member of the opposite sex in an attempt to gain a non-reproductive resource, irrespective of whether that attack is successful, the defending individual is likely to avoid or repel any subsequent interactions, thus reducing the probability of copulation. The non-reproductive resources gained via such agonistic intersex interactions may however increase future reproductive success, for instance by winning a territory (Wickman 1985; Bergman et al. 2007). In these circumstances the fitness benefits of immediate mating might be outweighed by the benefits of increased future reproductive success (Real 1990; Ah-King and Gowaty 2016). Furthermore, in such a scenario only individuals that would accrue fitness benefits from gaining the non-reproductive resource are likely to attack, as those already with the specific resource in question (e.g. territory or anti-predator defense) have lower intrinsic RV (Clark 1994), shifting the balance of costs and benefits to favor mating over fighting.

It is worth noting however, that even if an individual would benefit from obtaining the resource (high RV) it may not necessarily be able to win a contest if it has a lower RHP than its opponent (but see also Figler et al. 1995; Gherardi 2006). This is particularly relevant for intersex interactions, as sexual dimorphism often leads to significant differences between the sexes in body size (Teder and Tammaru 2005) and thus RHP (Colella et al. 2019). Even small differences in RHP have been shown to be important in determining the outcome of contests (Vieira and Peixoto 2013). Depending on how individuals assess their RHP, i.e. whether independent of or in relation to their opponent (Chapin et al. 2019), RHP differences might also directly feedback into an individual’s decision on whether to mate or fight. Therefore, a number of different and potentially interacting factors may contribute to the outcome of intersex interactions between potential contest and mating partners.

Here we investigate intersex interactions in the turnip sawfly *Athalia rosae* (Hymenoptera: Tenthredinidae). Female and male adults of *A. rosae* collect clerodane diterpene compounds, from now on called clerodanoids, from non-food plants (Nishida et al. 2004) which are then deposited on the cuticle (Amano et al. 1999). Clerodanoids act as a deterrent to predators (Nishida and Fukami 1990; Amano et al. 1999) and are known for their antimicrobial activity (Bozov et al. 2015). As such they represent a significant non-reproductive resource and adults fight each other to gain access to them, gathering clerodanoids via aggressive and energetically expensive nibbling on the exterior of conspecifics (preprint Paul et al. 2021), potentially lowering the defender’s chemical defense levels. The mating success of *A. rosae* females, but not that of males, has also been shown to be increased by the possession of clerodanoids (Amano et al. 1999). This has been suggested to indicate a potential role of clerodanoids as a female sex pheromone, however an alternative explanation is that this difference in mating success may be driven by fighting behavior between individuals. Female *A. rosae* are larger and often up to twice as heavy as males (Bandeili and Müller 2010; Paul et al. 2019) and so males with clerodanoids (C+) may be unable to mate with females without clerodanoids (C–), because the latter are able to aggressively nibble on C+ males due to their higher RHP. Finally, the need to copulate also differs between sexes in this haplodiploid species, as females are able to produce haploid male offspring (without mating) whereas males must copulate to reproduce (Naito and Suzuki 1991).

**Figure 1:**
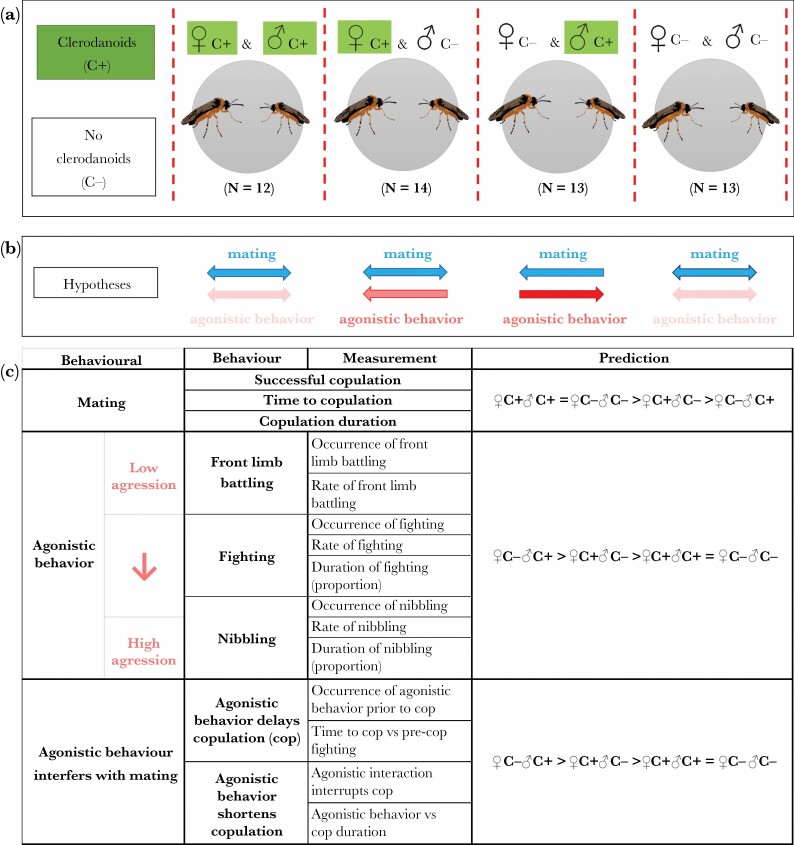
(A) Outline of two-way factorial experimental design showing the four different treatment levels and number (*N*) of *Athalia rosae* pairs used in the behavioral assays. Green boxes denote those individuals who had access to clerodanoids via nibbling on *Ajuga reptans* leaves (C+), those without green boxes had no access to *A. reptans* leaves and therefore do not have clerodanoids (C−). (B) Illustration of the general hypotheses for each treatment with the saturation and direction of each arrow representing the likelihood of individuals being motivated to display a certain behavior. (C) A full list of the behaviors measured and parameters tested alongside specific predictions for each set of parameters (see Supplementary Material S2 and methods for further information on each behavior and the parameters respectively).

By recording the mating and agonistic behavior of *A. rosae* female-male pairs with either symmetrical or asymmetrical clerodanoid acquisition (♀C+♂C+, ♀C+♂C−, ♀C−♂C+, ♀C−♂C−; Figure 1A) we aim to establish the degree to which these behaviors are influenced by clerodanoids (Figure 1B). The parameters measured fall into three main categories: mating, agonistic behavior, and agonistic behavior interferes with mating (Figure 1C). More generally our hypothesis is that both the clerodanoid status and sex of *A. rosae* will influence the degree to which mating and agonistic behavior occurs. Specifically we predict that: 1) Under symmetrical conditions (♀C+♂C+ & ♀C−♂C−) copulation will be more likely to occur, occur more rapidly, and last longer and that agonistic behavior (front limb battling, fighting, and nibbling) will occur less frequently, and if it does occur be briefer and therefore interfere less with mating behavior, than in asymmetrical treatment levels (♀C+♂C−, ♀C−♂C+); 2) Agonistic behavior will occur more frequently in asymmetrical treatment levels when females lack clerodanoids (♀C−♂C+) than when males lack them (♀C+♂C−) due to the larger size (proxy of RHP) of females than males; 3) The higher level of agonistic interactions in ♀C−♂C+ and the greater need of males than females to copulate will lead to a higher disruption of copulation in this treatment level than in ♀C+♂C−, both delaying copulation onset and interrupting its progress.

## METHODS

### Behavioral assays

Adult F2 *A. rosae* (6–13 days post eclosion; see Supplementary Material S1 for rearing details) were kept at ~5 ^o^C in a refrigerator upon emergence until use in behavioral assays. This prolonged the period over which the experimental work could be carried out, extending longevity from 20 days at room temperature (Bandeili and Müller 2010) to 3 months. Adults were removed from the refrigerator 48 h prior to the commencement of the behavioral assays. In order to overcome any difference in the behavior of the first mating of virgin males (Torres-Vila and Jennions 2005), males were mated once to a non-focal female without access to clerodanoids 48 h prior to the mating assay. Then, all adults were weighed to the nearest 0.01 mg (Sartorius AZ64, M-POWER Series Analytical Balance, Germany) and provided with a honey–water mixture. C+ individuals were additionally provided with a small section (1 cm^2^) of a leaf of *Ajuga reptans* (Lamiaceae) for 48 h, giving them the opportunity to incorporate clerodanoids prior to the start of the trial. Plants of *A. reptans* were collected from a population at the edge of a local forest (52°01′58.2″N 8°29′04.5″E) in the summer of 2018. This plant species does not serve as food plant but is used by *A. rosae* to gather clerodanoids (Nishida et al., 2004). Individuals that either were or were not exposed to *A. reptans* (i.e. C+ or C−) were handled with different forceps and forceps were cleaned with 70% ethanol in between each use to prevent an inadvertent transfer of chemical compounds between individuals. Assays were carried out between male and female *A. rosae* across four different treatment levels (♀C+♂C+, ♀C+♂C−, ♀C−♂C+, ♀C−♂C−; Figure 1A) set up to investigate the effects of clerodanoid access on mating and agonistic behaviors (Supplementary Material S2 for classification of each behavioral parameter). Due to the importance of size and age in determining outcome in dyadic contests (e.g. Humphries et al. 2006), pairs were set up in a way that minimized age and size differences across treatments (Supplementary Material S3). For each of the four treatment levels, an individual female was first added to a mating arena, consisting of a Petri dish (60 mm × 15 mm), and one male was then added to the opposite side of the arena (*N* = 12–14 replicates per treatment level, Figure 1A). Interactions were recorded for 25 min or until a single copulation had finished [*A. rosae* mate multiple times at regular intervals (Sawa et al. 1989)], using a Sony HDR-CX410VE camcorder (SONY EUROPE B.V.) (AVCHD – 1920 × 1080 − 25 fps). Video data was analyzed blind by the same observer using the software BORIS v 7.9.8 (Friard and Gamba 2016). All recorded agonistic contact and mating behavior was observed at 0.3× the original speed and at 2× display magnification to ensure that each behavior was categorized and timed correctly.

### Statistical analysis

All data were analyzed using R version 4.0.2 (R Core Team, 22 June 2020). Alpha level was set at 0.05 for all tests and model residuals were checked for normality and variance homogeneity. Models of count data were all tested for both zero-inflation (Hartig 2020) and overdispersion, and models were chosen accordingly. Likelihood ratio tests were employed to establish significance of main treatment effect. Posthoc analyses were carried out using “multcomp” v. 1.4-13 (Hothorn et al. 2016).

#### Mating

Variation in whether copulation occurred was assessed using a binomial model (package: “MASS” v. 7.3-51.6) with copulation occurrence as the response variable and treatment as the predictor variable. The effect of treatment on the time taken for copulation to commence and copulation duration was assessed using a linear model (lm, package: “MASS”), in which either log[time to copulation (s)] or copulation duration (s) was the response variable and treatment the predictor variable.

#### Agonistic behavior

Variation in the occurrence of leg battling, fighting, and successful nibbling across treatment levels (predictor) was assessed using individual binomial models, fitted using maximum penalized likelihood to handle separation when it occurred (package: “brglm”; Kosmidis and Firth 2020). The effect of treatment on the rate (occurrence per second) of leg battling, fighting, or successful nibbling was assessed using individual negative binomial generalized linear models (glm.nb, package: MASS). Each of these three response variables was modelled separately with treatment as the predictor variable and log[assay duration (s)] as an offset. How the proportion of time during the assay that females spent engaging in fighting or total successful nibbling varied depending on treatment (predictor) was modelled using individual beta regressions (package: “betareg”, Cribari-Neto and Zeileis 2010). The identity of the successful nibblers within a pair was established using a binomial model (package: “MASS”), where nibbling occurrence was the response variable and clerodanoid exposure, sex, their interaction, and pair ID were the predictors. PairID was set as a fixed opposed to random effect to avoid model overfitting. It is worth noting at this point that unlike many contest interactions, in which there is one winner or loser, here the successful nibbling of one individual on another does not preclude reciprocal nibbling from the other individual in a pair. Thus, analyses taking into account both individuals in a pair is valid (Briffa et al., 2013).

#### Agonistic behavior interferes with mating

Variation in the occurrence of agonistic behavior pre-copulation or across the end of copulation (i.e. copulation interrupted by agonistic interaction) across treatment levels (predictor) was assessed using individual binomial models, fitted using maximum penalized likelihood to handle separation when it occurred (packages: “MASS” or “brglm”). An lm (package: “MASS”) was used to assess both how the time until copulation (response) was influenced by whether agonistic behavior occurred prior to copulation (predictor) and whether copulation duration (response) was influenced by whether copulation ended with an agonistic interaction.

## RESULTS

### Mating

**Figure 2:**
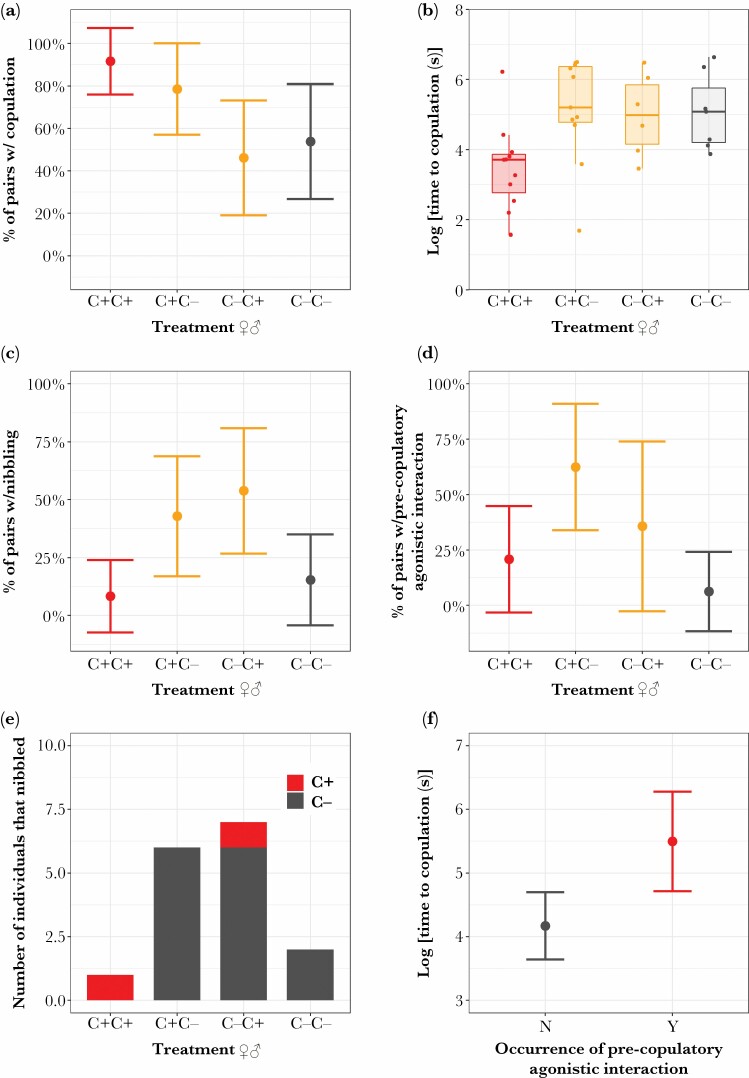
Effect of clerodanoid access (C+ = nibbled on *Ajuga reptans* leaf, C− = not nibbled on *A. reptans* leaf) on (A) the occurrence of successful copulation, (B) log time until copulation commenced, (C) the occurrence of successful nibbling, (D) whether fighting occurred before copulation, (E) the number of individuals that successfully nibbled their opponent split within treatment by whether or not they had clerodanoid access, and (F) time taken until copulation commenced depending on whether pre-copulatory agonistic behavior also occurred in adult *A. rosae.* Treatment levels are listed with females on the left and males on the right. Boxes in box plot show the median, the first and third quartiles (the 25th and 75th percentiles) at the hinge, and the whiskers extend to the largest or smallest value no further than 1.5 × IQR from the hinge for the upper and lower whiskers, respectively. Points with error bars in other plots are model predictions with 95% confidence intervals.

There was a significant effect of treatment on the number of pairs in which successful copulation occurred (*X*^2^_3,48_ = 8.40, *P* = 0.038), with the number of pairs copulating appearing to be highest in ♀C+♂C+ and lowest in ♀C−♂C+ (Figure 2A), although not significantly so (Supplementary Material S4A). For those pairs in which successful copulation occurred there was no significant effect of treatment on copulation duration (*F*_3,31_ = 1.19, *P* = 0.331). Time until copulation commenced was significantly affected by treatment (*F*_3,31_ = 3.92, *P* = 0.018), with individuals in the ♀C+♂C+ being the quickest to mate (Figures 2B and Supplementary Material S4B).

### Agonistic behavior

There was no effect of treatment on the occurrence (*X*^2^_3,48_ = 5.06, *P* = 0.167) or the rate of leg battling (*X*^2^_3,48_ = 3.32, *P* = 0.345) or on the occurrence (*X*^2^_3,48_ = 2.71, *P* = 0.437), rate (*X*^2^_3,48_ = 2.26, *P* = 0.520), or duration (pseudo-*R*^2^ = 0.113, (LRT) *X*^2^ = 4.11, df = 3, *P* = 0.249) of fighting. However, the occurrence of successful nibbling events did vary significantly with treatment (*X*^2^_3,48_ = 9.08, *P* = 0.028), a difference that appears to have been driven by pairs with asymmetrical clerodanoid access (Figure 2C) although not significantly so (Supplementary Material S4C), and the rate of successful nibbling was borderline (rate: *X*^2^_3,48_ = 7.37, *P* = 0.061). The successful nibbling of conspecifics was predominantly driven by C− individuals (*X*^2^_22,51_ = 21.94, *P* < 0.001, Figure 2E). In contrast there was no effect of treatment on nibbling duration (pseudo-*R*^2^ = 0.120, (LRT) *X*^2^ = 1.55, df = 3, *P* = 0.670).

### Agonistic behavior interferes with mating

The likelihood of having an agonistic interaction pre-copulation was significantly affected by treatment (*X*^2^_3,31_ = 10.12, *P* = 0.018), a difference that appears to have been driven by pairs in the ♀C+♂C− treatment level (Figure 2D) although not significantly so (Supplementary Material S4D). Time until copulation commenced was also longer in pairs where pre-copulatory fighting occurred (*F*_1,33_ = 7.57, *P* = 0.010, Figure 2F), whereas the interruption of copulation by agonistic behavior was common across all treatment levels (*X*^2^_3,31_ = 1.87, *P* = 0.600), and did not significantly influence copulation duration (*F*_1,33_ = 0.38, *P* = 0.544).

## DISCUSSION

The main aim of this study was to investigate the degree to which access to clerodanoids influences both mating and fighting behavior in *A. rosae.* Copulation patterns mirrored some but not all of our predictions, with pairs in the ♀C+♂C+ treatment mating more quickly and showing a trend for a higher likelihood of mating than pairs in the other three treatment levels. Furthermore, in contrast to our predictions, of the three agonistic behaviors measured (front limb battling, fighting, and nibbling) only successful nibbling varied significantly according to treatment, with the trend for higher nibbling in asymmetrical treatments being driven by C− individuals. We also found no evidence for a difference between the two asymmetrical treatments in agonistic behavior including successful nibbling, which we had predicted based on differences in size, and therefore RHP, of the C+ and C− individuals in each of the two treatments (♀C+♂C−, ♀C–♂C+). Nibbling did not occur more frequently in asymmetrical treatment levels where females lacked clerodanoids (♀C−♂C+) than when males lacked them (♀C+♂C−) despite the larger size (proxy of RHP) of females than males. As we discuss in detail below these results indicate an interplay between fighting and mating behavior in *A. rosae* that is subtly influenced by clerodanoids.

That the likelihood of copulation occurring was not inversely mirrored by patterns of any of the agonistic behaviors measured indicates that despite agonistic behavior delaying the onset of copulation it did not cost aggressors a mating opportunity. The probability of individuals mating despite agonistic behavior may have been influenced by the motivation of both males and females to mate. *A. rosae* males need to mate with females to reproduce (Naito and Suzuki 1991) and so the likelihood of their continuing to try to mate despite female agonistic behavior is high, not least because all experimental females were virgins which in some species are particularly attractive to males (e.g. King et al. 2005). In comparison, although *A. rosae* females can produce male offspring without mating (Naito and Suzuki 1991), depending on the operational sex ratio there can be a fitness benefit to producing females and therefore to mating (Godfray 1990; Boulton et al. 2015) . Furthermore, theory suggests that in some situations virgin females are less choosy than mated females (Kokko and Mappes 2005) Although much of this theory is based on diploid systems and *A. rosae* are haplodiploid, in non-eusocial haplodiploids such as parasitoid wasps there is mounting evidence that virgin females are fairly indiscriminate when it comes to mate choice (reviewed in Boulton et al., 2015) and preferentially search for males over oviposition sites (Steiner and Ruther 2009). Finally, the patterns of copulation observed here may not necessarily hold true in the wild, at least to the same extent. Mating experiments in another haplodiploid species (*Alabagrus texanus*) have shown increased rates of mating for virgin females in laboratory set ups, such as those used here, when compared to larger less enclosed spaces (Adams and Morse 2014).

Although conflict over clerodanoids does not seem to have cost individuals a mating opportunity it still disrupted mating behavior to a certain extent, as copulation in pairs with pre-copulatory conflict was significantly delayed. Such a delay appeared to be most prevalent when females had clerodanoids, but males did not (♀C+♂C−). Therefore, despite the potential benefits of mating, especially for males, individuals appear to have been willing to forgo immediate mating in order to acquire clerodanoids. This supports the idea that doing so may have significant fitness benefits for males by prolonging survival, via clerodanoid mediated protection against predators (Nishida and Fukami 1990; Amano et al. 1999) and disease (Bozov et al. 2015), and therefore increasing potential future mating opportunities. It is also worth noting that in general agonistic behavior between individuals was prevalent across all treatment levels, potentially indicating a degree of sexual conflict independent of clerodanoids, which warrants further investigation. Such agonistic behavior may be expressed to assess a partner’s quality or to avoid costly copulations (Arnqvist 1992) and the parallels between mating and fighting behavior in this regard are becoming increasingly apparent (Lane and Briffa 2021).

Despite the trend for a greater likelihood of nibbling occurring in both asymmetrical treatments we had anticipated that the substantial differences in body mass (Bandeili and Müller 2010; Paul et al. 2019) and therefore RHP between females and males would lead to higher successful nibbling in ♀C−♂C+ than in ♀C+♂C− pairs. The lack of such an observable pattern in our data could be due to a number of factors. Firstly, RHP is not the only determinant of contest outcome, an individual’s motivation (RV), dictated by the importance of a specific resource, also plays an important role (Humphries et al. 2006; Gherardi 2006). Such motivation can sometimes have a larger impact on contest outcome than RHP (e.g. Mohamad et al. 2010; Zhang et al. 2019) as may have been the case here with the motivation of C− males to gain clerodanoids being strong enough to overcome the size difference between them and the defending C+ female. It is worth noting however that such RV effects occur frequently only when size differences between competitors are small (e.g. O’Connor et al. 2015).

An additional explanation could be that we did not select the most exact measure of RHP in this study. Although a wealth of literature has demonstrated how disparities in size can determine the outcome of agonistic interactions and therefore represent good proxies for RHP (Briffa 2008, 2013; Chamorro-Florescano et al. 2011) it is by no means the only or the most important RHP determinant. Factors including skill (Briffa and Lane 2017), aggression (Favati et al. 2021), physiological traits (Briffa and Sneddon 2007), and personality (Rudin and Briffa 2012) can all contribute to an individual’s RHP. Furthermore, some of these RHP components can vary significantly between the sexes and independently of size, as is the case for aggression in stalk-eyed flies (*Teleopsis dalmanni*: Bubak et al. 2016; Bubak et al. 2019). This means that factors crucial for determining RHP in intrasex interactions may differ from those contributing to RHP and deciding contest outcome in intersex interactions. For example, if male *A. rosae* are more aggressive than females (as in *T. dalmanni*) or alternatively have a higher innate level of skill in contests, this may minimize RHP differences between males and females despite the larger size of the latter, leading to the similar levels of nibbling behavior observed here in both of the asymmetrical treatments. Such conjectures regarding these traits remain to be tested in *A. rosae,* but our results highlight the complexity of predicting which phenotypic variables are the most important determinants of RHP in intersex interactions.

The trend for higher copulation success in those treatments where females had clerodanoids (♀C+♂C+, ♀C+♂C−) suggests that the impact of clerodanoids on mating behavior may not just be restricted to its delay via agonistic behavior. Previous work has indicated that male *A. rosae* prefer C+ females but that females show no preference for C+ males (Amano et al. 1999). Through their positive effect on female survival, with better protection against predatory birds and lizards (Nishida and Fukami 1990; Amano et al. 1999), clerodanoids could arguably provide a reliable binary signal of female quality. The higher survival of C+ females compared to C− females, due to their lower predation risk, leading to a potentially larger number of eggs oviposited (increasing fitness). Although speculative, such a dual role of clerodanoids would fit the general pattern of infochemical flexibility observed in insects (Weiss et al. 2013; Cheng et al. 2017) and perhaps explain the previously identified higher mating success of C+ females. However, unlike the previous study we did not use a mate choice assay, so support for clerodanoids playing such a role in signaling female quality is far from conclusive. Neither are both explanations—mate attraction versus conflict over defense compounds—mutually exclusive, both could be contributing to the behavior observed in this experiment.

In summary, we investigated agonistic intersex interactions over a non-reproductive resource between potential mating partners and to our knowledge show for the first time that fighting over such a resource can have knock-on effects for the onset of mating. The success of aggressive nibbling behavior in pairs with asymmetrical clerodanoid exposure was not influenced by a superiority of mass in attacking C– individuals (as determined by sexual dimorphism), adding to the growing body of evidence that size differences are not always the most important determinant of RHP. Further work is needed to establish factors other than size that may contribute to RHP in both male and female *A. rosae* and the effect that variation in these traits has on female-male interactions such as the ones outlined here. We also suggest that the importance of traits determining RHP may differ between intrasex and intersex interactions in the same species. Finally, what remains unknown is how such agonistic behavior over non-reproductive resources may have shaped the evolution of sexual traits in this species. For example, do females prioritize clerodanoid acquisition more than males (sensory bias) if it increases male attraction and therefore mate choice for females? Overall, we demonstrate the importance and potential of studying agonistic intersex interactions over non-reproductive resources and hope that this work stimulates further research in the area.

## Supplementary Material

arab117_suppl_Supplementary_S1

arab117_suppl_Supplementary_S2

arab117_suppl_Supplementary_S2a

arab117_suppl_Supplementary_S2b

arab117_suppl_Supplementary_S3

arab117_suppl_Supplementary_S4

## Data Availability

Analyses reported in this article can be reproduced using the data and code provided by Paul and Müller (2021).
