# Supplementary material for: Fighting over defense chemicals disrupts mating behavior
Source: Behav Ecol. 2021 Dec 31;33(2):329–35. doi: 10.1093/beheco/arab117 (PMC9015217; doi:10.1093/beheco/arab117)
Supplement: arab117_suppl_Supplementary_S1 [file arab117_suppl_supplementary_s1.docx]

**S1. Experimental rearing of sawflies (F0-F2):** Sixty adults of *A. rosae* (F0) were collected in a meadow in Verl, Germany (51°52'23.0"N 8°33'32.0"E) in July 2018 and split into two breeding groups, A and B, placed each in a mesh cage (60 x 60 x 60 cm). Females in each cage were provided with plants of *Sinapis alba* (Brassicaceae) for egg laying and emerging F1 larvae were provided with plants of *Brassica rapa* var. *pekinensis* (Brassicaceae) as food. Larvae of the last instar (eonypmhs) were placed in individual pots containing ~30 g sterilised soil for pupation and after emergence F1 females were mated with an F1 male from the opposite breeding group (e.g. F1♀A x F1♂B). Post mating each mated F1 female (N=30, produce male and female offspring) and each virgin female [N=30, produce only male offspring (Naito & Suzuki, 1991)] were given an individual *S. alba* plant for oviposition and provided *ad libitum* with a honey-water mixture (1:50). Emerging F2 larvae were collected daily from each plant and the larvae were reared in ventilated containers (25 cm x 15 cm x 10 cm). Larvae were provided with *ad libitum* middle-aged *B. rapa* leaves and moistened tissue paper to prevent desiccation. Eonymphs were placed individually in soil pots for pupation and after emergence F2 adults were housed individually in Petri dishes (35 mm) with honey water-infused tissue paper as a food supply. These adults were kept at ~5 ^o^C in a refrigerator until use in behavioral assays, which due to their short life span prolonged the period over which the experimental work could be carried out. All rearing was carried out in a climate chamber [temp: 20 °C:16 ^o^C (16 h:8 h), light:dark (16 h:8 h), 70 % r.h.] and all host plants were grown from seeds in a greenhouse (no climate control, light:dark 16 h:8 h).
