# Supplementary material for: Fighting over defense chemicals disrupts mating behavior
Source: Behav Ecol. 2021 Dec 31;33(2):329–35. doi: 10.1093/beheco/arab117 (PMC9015217; doi:10.1093/beheco/arab117)
Supplement: arab117_suppl_Supplementary_S2 [file arab117_suppl_supplementary_s2.docx]

**S2.** Behaviors recorded during mating trials of *Athalia rosae* with descriptions of each behavior. Behaviors are divided into agonistic behavior and mating behavior. State behaviors have their occurrence and duration recorded and point behaviors their occurrence but not duration (see S2 A & B for example videos).

| **Behavioral code** | **Behavioral type** | **Description** | |
| --- | --- | --- | --- |
| **Mating behavior** | | | |
| **Attempted copulation** | Point | Male moves/flies towards female with curved abdomen, but with no attempted nibbling (movement of head towards thorax/abdomen/wings/head). | |
| **Copulation** | State | Copulation initiated. Start of copulation coded as the moment that the genitals become attached (male inserts genitalia into female). When mating occurs individuals often become still, facing in opposite directions with their wings erect, however if they are fighting at the start of copulation this does not always happen immediately, therefore though this posture is indicative of mating, it does not necessarily indicate the correct start of copulation. | |
| **Walking during copulation** | State | Female-specific behavior. Classified as the movement of a female during copulation, for a distance of at least half a body length in a forward direction. | |
| **Agonistic contact behavior** | | | |
| **Front limb battling** | State | Both individuals face each other and use front legs in ‘battling’ motion. Front legs make contact and both individuals engage in behavior. | |
| **Fighting / attempted nibbling** | State | Individuals use all legs and move entire body when fighting. Distinguished from nibbling by a lack of contact of mouthparts with the body of the other individual. | |
| **Successful nibbling** | State | Mouthparts of attacker make contact with the body of the defender – individual-specific behavior. Duration of nibbling for each individual based on the criteria of mouthpart contact. | |
| **Agonistic behavior prior to copulation** | Point | ‘Successful nibbling' and/or 'Fighting/attempted nibbling' (i.e. an antagonistic interaction) before mating was classified as any of these behaviors that started before mating occurred; it did not have to finish before mating also occurred. | |
| **Agonistic behavior ends copulation** | Point | Successful nibbling' and/or 'Fighting/attempted nibbling' (i.e. an antagonistic interaction) starting before the end of copulation and ending after the end of copulation. |  |
