# Supplementary material for: Fighting over defense chemicals disrupts mating behavior
Source: Behav Ecol. 2021 Dec 31;33(2):329–35. doi: 10.1093/beheco/arab117 (PMC9015217; doi:10.1093/beheco/arab117)

**S3. Mass and age across treatment levels**

Female mass (g), male mass (g), mass difference between males and females (g), female age (days), and male age (days) were each assessed using a linear model (LM) (package: ‘MASS’ v 7.3-51.6), in which treatment was the predictor variable and female mass, male mass, mass difference, female age, and male age were the response variables for each model. There was no significant effect of treatment on female mass (F = 1.62, df = 3, 48, p = 0.198; Fig S3a), male mass (F = 0.52, df = 3, 48, p = 0.667), mass difference between the sexes (♀ mass - ♂ mass: F = 2.11, df = 3, 48, p = 0.111; Fig S3b), female age (F = 0.33, df = 3, 48, p = 0.807; Fig S3c), or male age (F = 0.56, df = 3, 48, p = 0.643; Fig S3d).


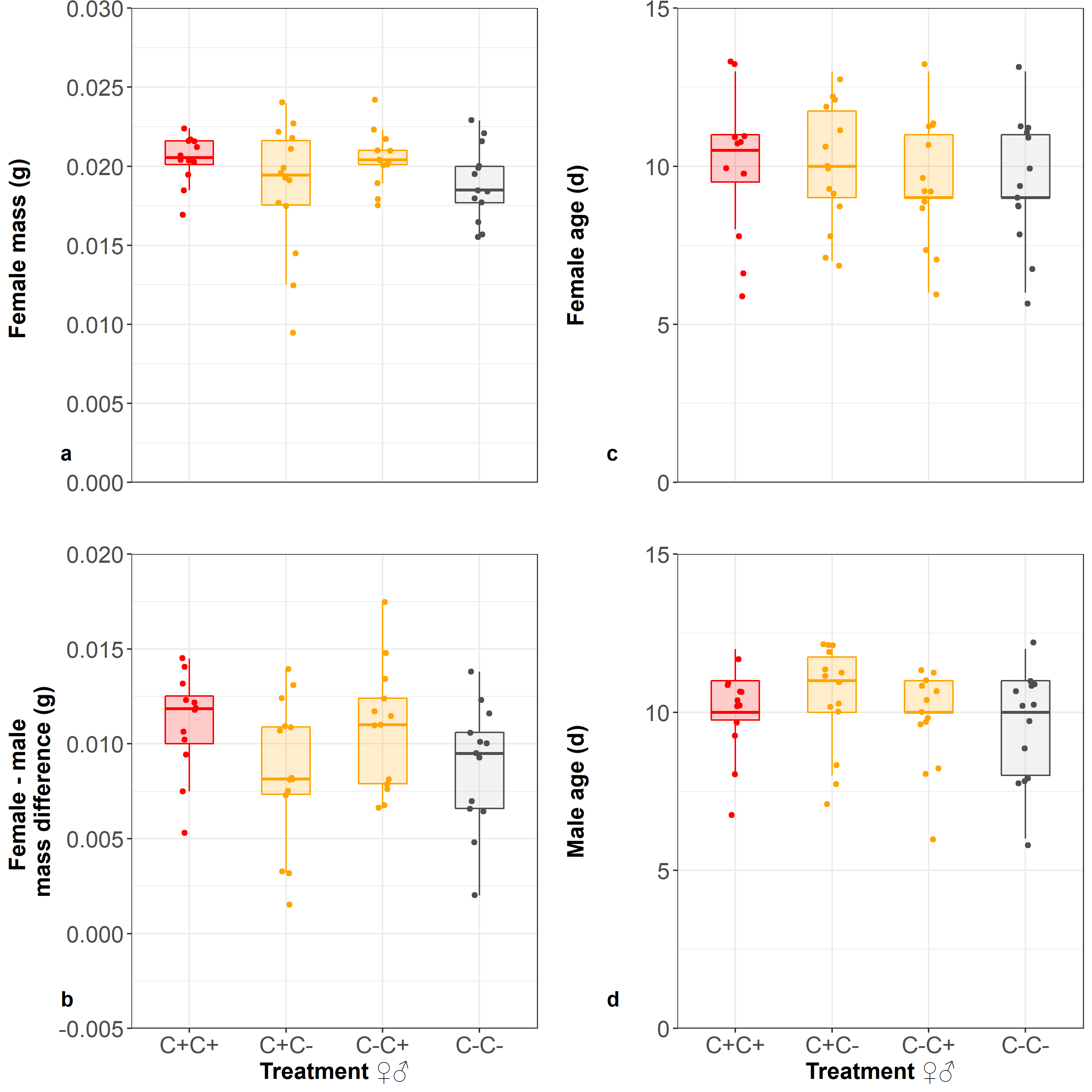

Supplement: arab117_suppl_Supplementary_S3 [file arab117_suppl_supplementary_s3.docx]
