# Supplementary material for: Fighting over defense chemicals disrupts mating behavior
Source: Behav Ecol. 2021 Dec 31;33(2):329–35. doi: 10.1093/beheco/arab117 (PMC9015217; doi:10.1093/beheco/arab117)
Supplement: arab117_suppl_Supplementary_S4 [file arab117_suppl_supplementary_s4.docx]

**S4**. Results of posthoc tests carried out using ‘multcomp’ package [v. 1.4-13 (Hothorn et al., 2016)] glht function. Pairwise comparisons carried out using ‘tukey contrasts’ and adjusted p-values reported (single step method).

**A. Copulation occurrence** (0/1). Fit: glm(formula = Copulation occurence ~ Treatment, family = binomial(link = "logit"), data = Dat, na.action = na.omit)

**B. Time until onset of copulation (s)**. Fit: glm(formula = log(Time to Copulation) ~ Treatment, family = gaussian, data = Dat, na.action = na.omit)

**C. Nibbling occurrence (0/1)**. Fit: glm(formula = Nibbling ~ Treatment, family = binomial(link = "logit"), data = Dat, na.action = na.omit)

**D. Occurrence of agonistic behaviour prior to copulation (0/1)**. Fit: brglm(formula = Precopulatory fighting ~ Treatment, family = binomial(link = "logit"), data = Dat, na.action = na.omit, model = TRUE, method = "brglm.fit")
